# Moses’ Regulations Concerning the Hygiene of Organs of Generation

**Published:** 1885-11

**Authors:** Noël Gueneau De Mussy


### MOSES' REGULATIONS CONCERNING THE HYGIENE OF ORGANS OF GENERATION.

Moses found the practice of circumcision established in his race since the time of Abraham, and directed its continuance. It has been stated that Abraham borrowed this custom from the Egyptians. The statement is thought to be supported by the authority of Herodotus, who had observed the custom in Egypt. But as stated by Dr. Bouisson (Dict. Encyclop. V, XVII), Herodotus wrote nine hundred years after Moses, and the Egyptians were probably inspired by the example of the Israelites, as is affirmed by several historians. The custom renders less easy the transmission of contagious maladies produced by sexual relations, and it is asserted by Israelites that the practice prevents those pruriginous affections which may become a cause of irritation and of vicious habits. These conditions, which are often dangerous, are extremely rare among them. In the hygiene of women there is a point of capital importance—the regularity of the catamenial function, which should be accomplished in calm repose. A large number of the affections appertaining to the utero-ovarian apparatus may be referred to imprudences committed during the menstrual period. The congestion which precedes and accompanies the periodic flux appears to be connected with ovulation. If the catamenia are, as has been stated, accouchements in miniature, we know that fatigue, strong emotion, and all excitements should be severely interdicted during this period. Nevertheless, the education and the exigencies of modern society take no account of this matter. Most women do not make any change in their habits; they take long promenades, dance, travel, and ride horseback quite as though they were in a normal condition. Moses, however, affirmed and enforced a proper respect for the catamenial period. During its continuance the woman was obliged to remain sequestrated.—Dr. Noël Gueneau de Mussy, in *L'Union Médicale.*

